# Bone Immune Response to Materials, Part II: Copper and Polyetheretherketone (PEEK) Compared to Titanium at 10 and 28 Days in Rabbit Tibia

**DOI:** 10.3390/jcm8060814

**Published:** 2019-06-07

**Authors:** Ricardo Trindade, Tomas Albrektsson, Silvia Galli, Zdenka Prgomet, Pentti Tengvall, Ann Wennerberg

**Affiliations:** 1 Department of Prosthodontics, Institute of Odontology, The Sahlgrenska Academy, University of Gothenburg, 405 30 Gothenburg, Sweden; ann.wennerberg@odontologi.gu.se; 2 Department of Biomaterials, Institute of Clinical Sciences, University of Gothenburg, 405 30 Gothenburg, Sweden; tomas.albrektsson@biomaterials.gu.se (T.A.); pentti.tengvall@gu.se (P.T.); 3 Department of Prosthodontics, Faculty of Odontology, Malmö University, 205 06 Malmö, Sweden; silvia.galli@mau.se; 4 Department of Oral Pathology, Faculty of Odontology, Malmö University, 205 06 Malmö, Sweden; zdenka.prgomet@mau.se

**Keywords:** biomaterial, bone, osseointegration, immune, implant, healing, titanium, PEEK, Cu

## Abstract

Osseointegration is likely the result of an immunologically driven bone reaction to materials such as titanium. Osseointegration has resulted in the clinical possibility to anchor oral implants in jaw bone tissue. However, the mechanisms behind bony anchorage are not fully understood and complications over a longer period of time have been reported. The current study aims at exploring possible differences between copper (Cu) and polyetheretherketone (PEEK) materials that do not osseointegrate, with osseointegrating cp titanium as control. The implants were placed in rabbit tibia and selected immune markers were evaluated at 10 and 28 days of follow-up. Cu and PEEK demonstrated at both time points a higher immune activation than cp titanium. Cu demonstrated distance osteogenesis due to a maintained proinflammatory environment over time, and PEEK failed to osseointegrate due to an immunologically defined preferential adipose tissue formation on its surface. The here presented results suggest the description of two different mechanisms for failed osseointegration, both of which are correlated to the immune system.

## 1. Introduction

Osseointegration [1] is a central event for oral implant function. This specific bone reaction has been described and studied at length for titanium and other materials. Technical innovations have led to improvements of bone reactions, such as material surface topographical changes [2,3,4] that have been vastly adopted by the oral implant industry, as well as different forms of chemical surface modulations [5,6]. Such surface related innovations have resulted in improved clinical results and widening of clinical indications [7,8]. However, the specific bone related control mechanisms that lead to osseointegration are still in need of scientific analyses, as are the reasons for marginal bone resorption. Generally speaking, the foreign body reaction (FBR) is accepted as the series of host events that follow the introduction of a material into tissues. The host–biomaterial interaction [9] depends on the type of material, clinical handling and on the tissue where the implant is placed (e.g., bone, skin, and blood vessel), as well as the host specific conditions. The immune system has a central role in the FBR [10,11,12] where the M1/M2-macrophage phenotype balance has been identified as one of the main controlling factors at the cellular level [13]. Macrophages are thus able to shift between an M1-phenotype (proinflammatory) and an M2-phenotype (reparative/anti-inflammatory), with obvious consequences for tissue reaction to biomaterials, and experimental modulation of this balance has been studied to direct a favorable pathway for bone regeneration [14]. The current authors have demonstrated an early M1/M2 shift around titanium, at 10 days of follow-up towards a dominant M2 macrophage phenotype [15], in contrast to other materials such as polyetheretherketone (PEEK) and Copper (Cu) that present mixed M1/M2 phenotypes at the same short term of follow-up. Osseointegration is thus seen as the result of an FBR which in the long run may achieve a foreign body equilibrium allowing for long term loading of implants [16]. However, the basis for the control of bone metabolism around implants in health and disease remains largely unclear [17]. Particularly the events taking place after the inflammatory period of initial healing and a possible immunological regulation of bone metabolism are examples of important fields for further studies. Our group has demonstrated that titanium activates the immune system when compared to a sham site at 10 and 28 days of follow-up [12]. In Part I of this series of studies (where the current work is Part II), the importance of the specific immune response around different materials when compared to a sham site was demonstrated at an early stage of 10 days [15]. The current study aims at comparing materials that do not osseointegrate, i.e., test materials copper (known to induce a pronounced FBR in soft tissues [18]) and PEEK (considered a bioinert material [19]), to a material that osseointegrates, cp titanium (control) at 10 and 28 days, in order to investigate and compare the respective immune modulation reactions between the inflammatory (10 days) and postinflammatory (28 days) stages of bone healing.

## 2. Materials and Methods

The current study consists of an experiment in the rabbit proximal tibia (metaphysis), comparing bone healing on sites where osteotomies were performed and one of three test materials were placed for comparison: titanium (Ti), copper (Cu), or polyether ether ketone (PEEK), where Ti was a control.

**Figure 1:**
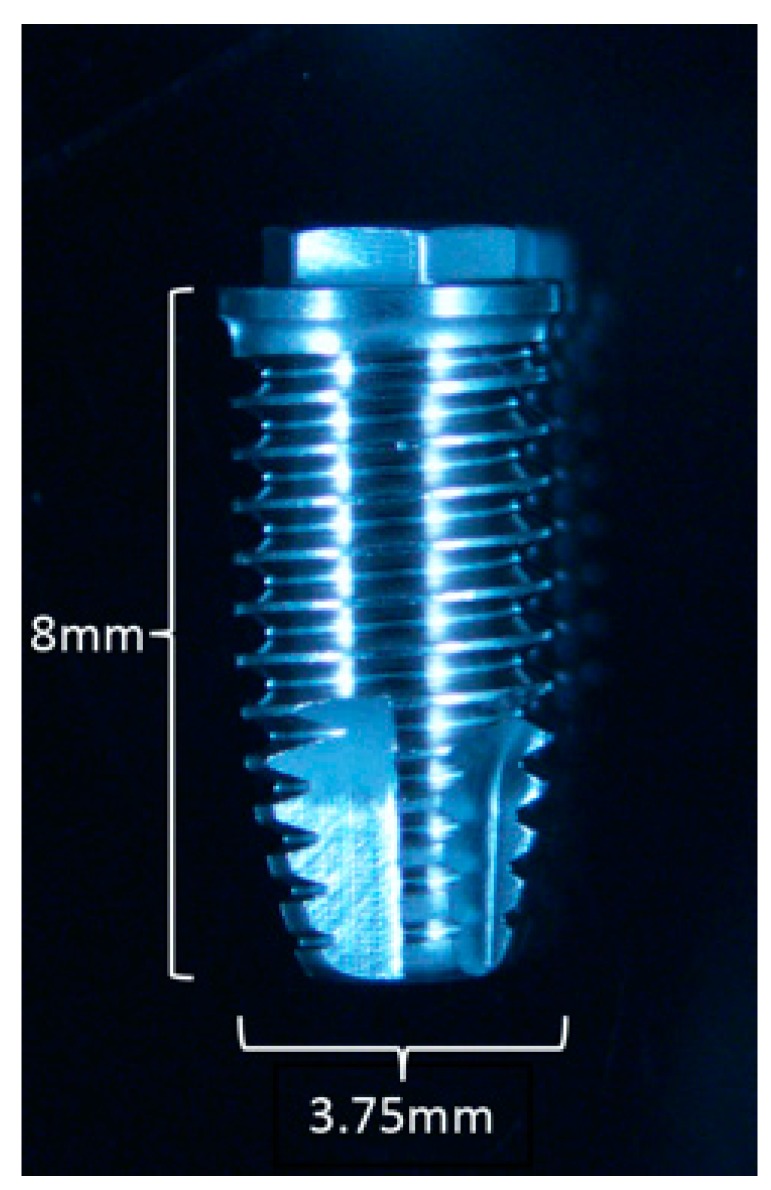
Implant design with 3.75mm width and 8mm length. Representative image of titanium (Ti) implant; copper (Cu) and polyetheretherketone (PEEK) implants with the same design.

All implants were turned with a threaded 0.6 mm pitch height, 3.75 mm width, and Branemark MkIII design (Figure 1). The Ti implants were made of commercially pure titanium grade IV (98.55% Ti, with specified maximum traces of elements Fe, O, N, H, and C for the remaining 1.45%).

### 2.1. Surgical Procedure

This study was performed on 12 mature, female New Zealand White Rabbits (*n* = 6 for each time point, 10 and 28 days, weight 3 to 4 Kg), with the ethical approval from the Ethics Committee for Animal Research (No. 13-011) of the École Nationale Vétérinaire D’Alfors, Maisons-Alfors, Val-de-Marne, France. The 6 animals at 10 days are the same used for Part I of this series of studies [12]. All care was taken to minimize animal pain or discomfort during and after the surgical procedures. For the surgical procedures, the rabbits were placed under general anesthesia using a mixture of medetomidine (Domitor; Zoetis, Florham Park, NJ, USA), ketamine (Imalgène 1000; Merial, Lyon, France), and diazepam (Valium; Roche, Basel, Switzerland) for induction, then applying subcutaneous buprenorphine (Buprecare; Animalcare, York, UK) and intramuscular meloxicam (Metacam; Boehringer Ingelheim Vetmedica, Inc., Ridgefield, CT, USA). A single incision was performed in the internal knee area on each side and the bone exposed for osteotomies and insertion of implants in the sites mentioned above. The surgical site was sutured with a resorbable suture (Vicryl 3/0; Ethicon, Cincinnati, OH, USA) and hemostasis achieved. Following surgery, Fentanyl patches (Duragesic; Janssen Pharmaceutica, Beerse, Belgium) were applied.

The osteotomies were produced with a sequence of increasing diameter twist drills, from 2 mm to 3.15 mm width, and a final countersink bur prepared the cortical part of the bone. The implants used were 3.75 mm in diameter, placed in an underprepared osteotomy to achieve primary (mechanical) stability.

The rabbits were housed in separate cages and were allowed to move and eat freely.

At 10 and 28 days, the rabbits were sacrificed with a lethal injection of sodium pentobarbital (Euthasol; Virbac, Fort Worth, TX, USA). The 6 animals at each time point had the implants removed through unscrewing. On 4 animals at 10 days and 5 animals at 28 days, bone was collected with a 2 mm twist drill from the periphery of the Ti, Cu, and PEEK sites on the most distal portion, and then processed through quantitative-polymerase chain reaction (qPCR). After this, at each time point, the implant sites were removed en bloc for histological processing on the 6 animals.

### 2.2. Gene Expression Analysis—qPCR

The bone samples for gene expression analysis at 10 or 28 days were collected from the distal side of the osteotomies of all three groups (following the removal of the implant from the implant sites), with a 2 mm twist drill that removed both cortical and marrow bone in the full length of the osteotomy, to enable the study of the 2 mm peri-implant bone area of each of the Ti, Cu, and PEEK sites. The samples were immediately transferred to separate sterile plastic recipients containing RNA*later* medium (AmbionInc, Austin, TX, USA) for preservation. The samples were then refrigerated first at 4 °C and then stored at –20 °C until processing.

#### 2.2.1. mRNA Isolation 

Samples were homogenized using an ultrasound homogenizer (Sonoplus HD3100, Brandelin) in 1 ml PureZOL and total RNA was isolated via column fractionalization using the Aurum^TM^ Total RNA Fatty and Fibrous Tissue Kit (Bio-Rad Laboratories Inc.; Hercules, CA, USA) following the manufacturer’s instructions. All the samples were DNAse treated using an on-column DNAse I contained in the kit to remove genomic DNA. The RNA quantity for each sample was analyzed in the NanoDrop 2000 Spectrophotometer (Thermo Scientific; Wilmington, DE, USA). BioRad iScript cDNA synthesis kit (Bio-Rad Laboratories Inc.; Hercules, CA, USA) was then used to convert mRNA into cDNA, following the manufacturer’s instructions.

qPCR primers (Tataa Biocenter; Gothenburg, Sweden) were designed following the NCBI Sequence database, including the local factors chosen in order to characterize the immune, inflammatory, and bone metabolic pathways (Table 1 and Table 2). All primers had efficiency between 90% and 110%.

#### 2.2.2. Amplification Process

Five microliters of SsoAdvanced SYBR^™^ Green Supermix (Bio-Rad Laboratories Inc.; Hercules, CA, USA) and 1 µL of cDNA template together with 0.4 µM of forward and reverse primer were used in the qPCR reaction. Each cDNA sample was performed on duplicates. The thermal cycles were performed on the CFX Connect Real-Time System (Bio-Rad Laboratories Inc.; Hercules, CA, USA). The CFX Manager Software 3.0 (Bio-Rad, Hercules, CA, USA) was used for the data analysis. 

Three genes (*GAPDH*, *ACT-beta*, and *LDHA*) were selected as reference genes using the geNorm algorithm integrated in the CFX Manager Software. A quantification cycle (Cq) value of the chosen reference genes (Table 1 and Table 2) was used as control; hence the mean Cq value of each target gene (Table 1) was normalized against the reference gene’s Cq, giving the gene’s relative expression. For calculation of fold-change, the ^ΔΔ^Cq was used, comparing mRNA expressions from the different groups. Significance was set at *p* < 0.05 and the regulation threshold at ×2 fold-change.

### 2.3. Decalcified Bone Histology

After removal of the implants from the studied Ti, Cu, and PEEK sites on the 6 subjects of each time point, bone was removed en bloc and preserved in 10% formalin (4% buffered formaldehyde; VWR international, Leuven, Belgium) during 48 h for fixation. Samples were decalcified in Ethylene diamine tetra-acetic acid (10% unbuffered EDTA; Milestone srl, BG, Italy) for 4 weeks, with weekly substitution of the EDTA solution, dehydrated and embedded in paraffin (Tissue-Tek TEC; Sakura Finetek Europe BV, Leiden, NL, USA). Samples were sectioned (4-µm-thick) with a microtome (Microm HM355S; Microm International GmbH, Thermo Fischer Scientific, Walldorf, Germany) and stained with Haematoxylin-Eosin (HE) for histological analysis.

### 2.4. Statistical Analysis

The gene expression statistical analysis was performed using the *t*-test built in the algorithm of the CFX Manager Software 3.0 package (BioRad, Hercules, CA, USA).

## 3. Results

### 3.1. Gene Expression Analysis

#### 3.1.1. Days

**Figure 2:**
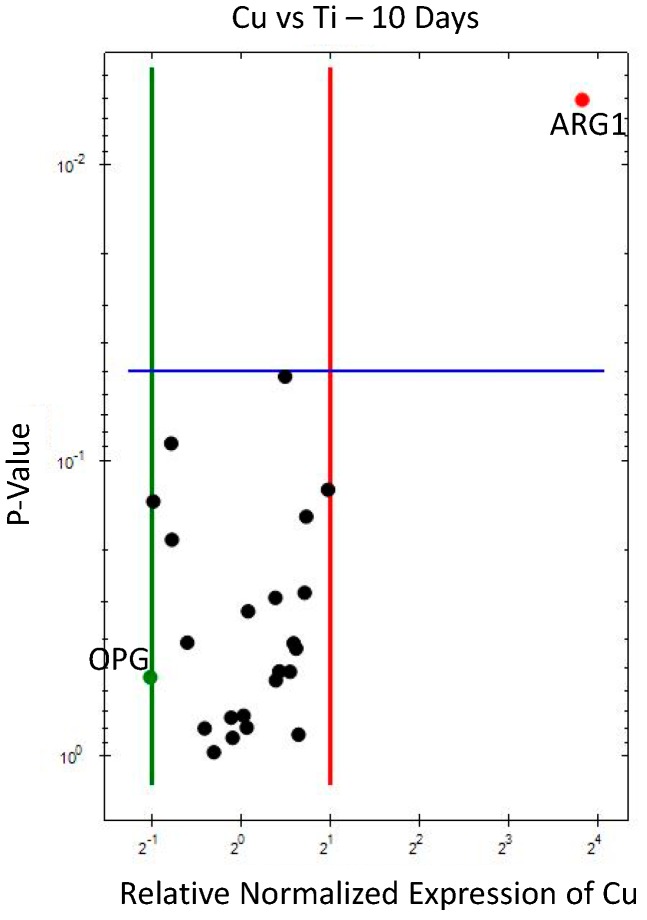
Volcano plot for gene expression of Cu compared to Ti (10 days). Downregulation (vertical green line) and upregulation (vertical red line) set at ×2 regulation. Statistical significance (set at *p* < 0.05) when marker above horizontal blue line.

**Figure 3:**
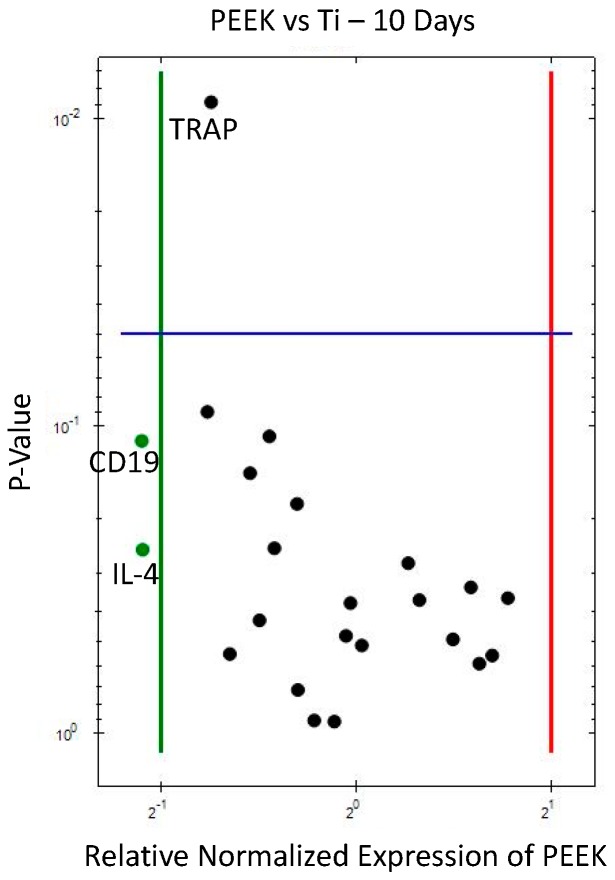
Volcano plot for gene expression of PEEK compared to Ti (10 days). Downregulation (vertical green line) and upregulation (vertical red line) set at ×2 regulation. Statistical significance (set at *p* < 0.05) when marker above horizontal blue line.

The gene expression analysis results at 10 days, comparing Cu and PEEK against Ti (control), are displayed in the volcano plots (Figure 2 and Figure 3) and data given in corresponding tables (Table 3 and Table 4) with the numerical results expressed in fold-change (regulation, x-axis) and significance (*p* value, y-axis). Data from 10 days have been published in Part I [15] of this study, if with another control.

At 10 days, Cu (vs. Ti, Figure 2 and Table 3) triggered an increased expression of *ARG1* gene (around 14× fold-change). This probably translates to a much higher presence of M2 macrophages (reparative phenotype) around Cu. *NCF1* showed close to a 2× fold upregulation for Cu, and elicited an increased participation of neutrophils at this early stage. Less increased markers, with approximately ×1.5 fold-change were observed for Complement (*C3aR1*), M1-macrophages (*CD14*) [20,21], B-lymphocytes (*CD19*) and Th/Treg-lymphocytes (*CD4*). On the other hand, Cu displayed a downregulation in *TRAP*, *PPAR-gamma* and *OPG*.

At 10 days PEEK (vs. Ti, Figure 3 and Table 4) showed less downregulation of the same bone remodeling markers as Cu, *TRAP*, *OPG*, and *PPAR-gamma*, as well as the B cell marker *CD19* and macrophage fusion marker *IL-4*. Increased expression of *NCF1* was observed for PEEK, probably translating to an increased presence of neutrophils around this material (as also observed for Cu).

#### 3.1.2. 28 Days

**Figure 4:**
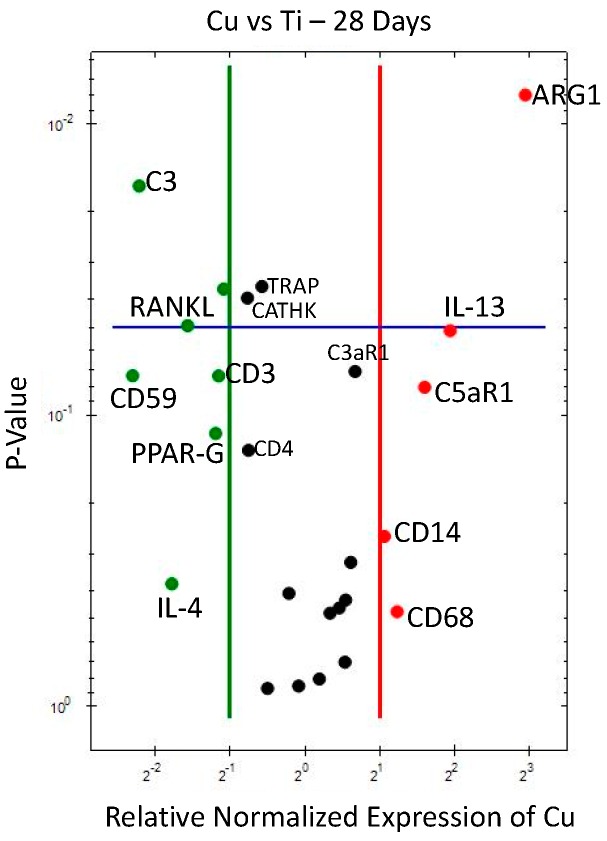
Volcano plot for gene expression analysis of Cu compared to Ti (28 days). Downregulation (vertical green line) and upregulation (vertical red line) set at ×2 regulation. Statistical significance (set at *p* < 0.05) when marker above horizontal blue line.

At 28 days, Cu against Ti (Figure 4 and Table 5), showed upregulation around Cu of *CD68* and *CD14*, as well as *ARG1* (macrophages of both M1-and M2- phenotypes), with M2 far more significant, indicating an overall higher macrophage activation for Cu vs. Ti at 28 days, when compared to 10 days. Complement markers *C3aR1* and *C5aR1* are also upregulated around Cu, as well as *IL-13* (a macrophage fusion marker). On the other hand, there was downregulation of bone remodeling markers *TRAP*, *CATHK*, *PPAR-gamma*, and *RANK*L, as well as *CD3* and *CD4* (T lymphocytes), *C3* complement factor, *CD59* (complement inhibitor), and *IL-4* (the other macrophage fusion marker). 

**Figure 5:**
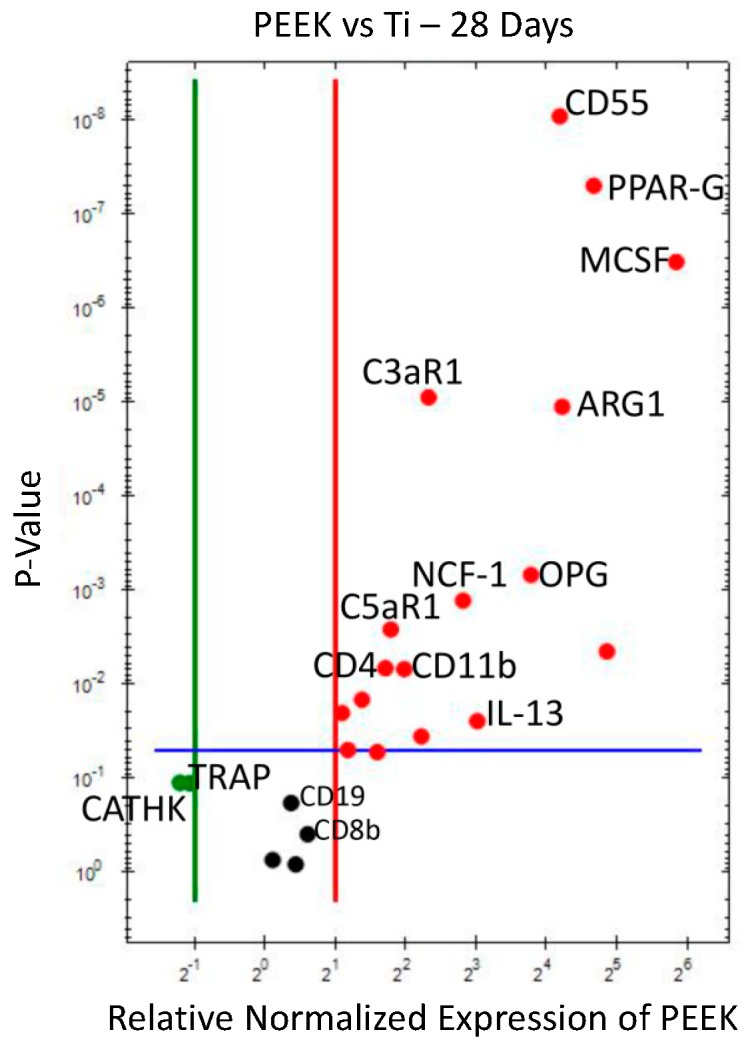
Volcano plot for gene expression analysis of PEEK compared to Ti (28 days). Only 2 subjects to be interpreted with caution. Downregulation (vertical green line) and upregulation (vertical red line) set at ×2 regulation. Statistical significance (set at *p* < 0.05) when marker above horizontal blue line.

PEEK vs. Ti at 28 days (Figure 5 and Table 6), showed upregulation of most markers used, with the exception of *TRAP* and *CATHK*, which are effector bone resorption markers that were downregulated. This excessive upregulation indicates a wide and strong immune activation around PEEK compared to Ti at 28 days. However, these results had a limitation in that only two out of the five rabbits used in the study allowed enough mRNA extraction on PEEK samples for gene expression analysis (see Discussion). Nevertheless, both subjects’ results were analysed separately for regulation (fold-change) and showed similar responses compatible with that presented in the overall results. However, the significance (*p* value) should not be taken in consideration here, since the low number of subjects (only 2) renders impossible a statistical analysis.

It is interesting to note that T_helper_/T_reg_ (*CD4*) was upregulated for Cu at 10 days, but downregulated at 28 days, which could indicate a shift in the presence of T lymphocytes between the two time points and these cells’ participation in the biomaterial-associated healing process.

### 3.2. Comparative Analysis of Gene Expression: 10 vs. 28 Days

**Figure 6:**
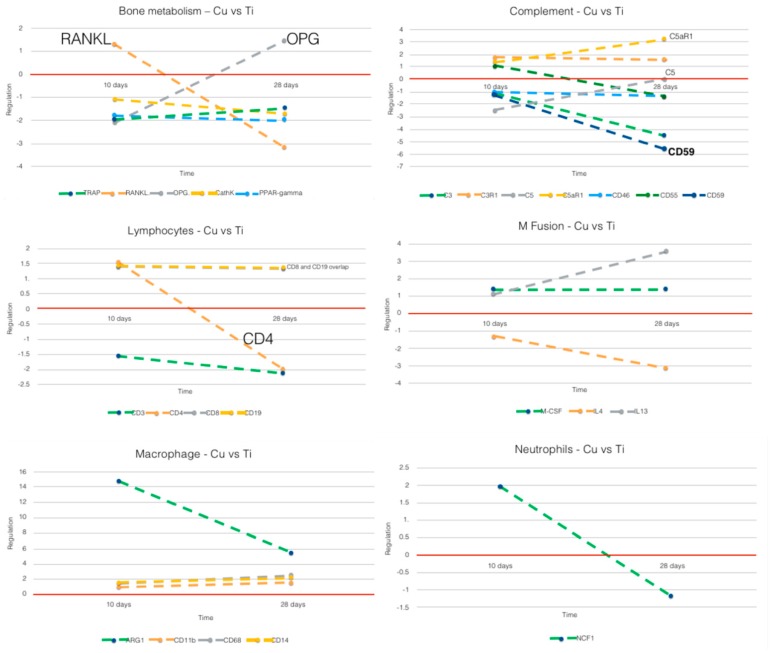
Comparative analysis of gene expression between 10 and 28 days for Cu vs. Ti. Horizontal red line: zero regulation mark; x-axis: time; y-axis: gene marker regulation (10 or 28 days). Intermittent lines do not represent actual results at time points other than 10 or 28 days, but only highlight trend from 10 to 28 days.

**Figure 7:**
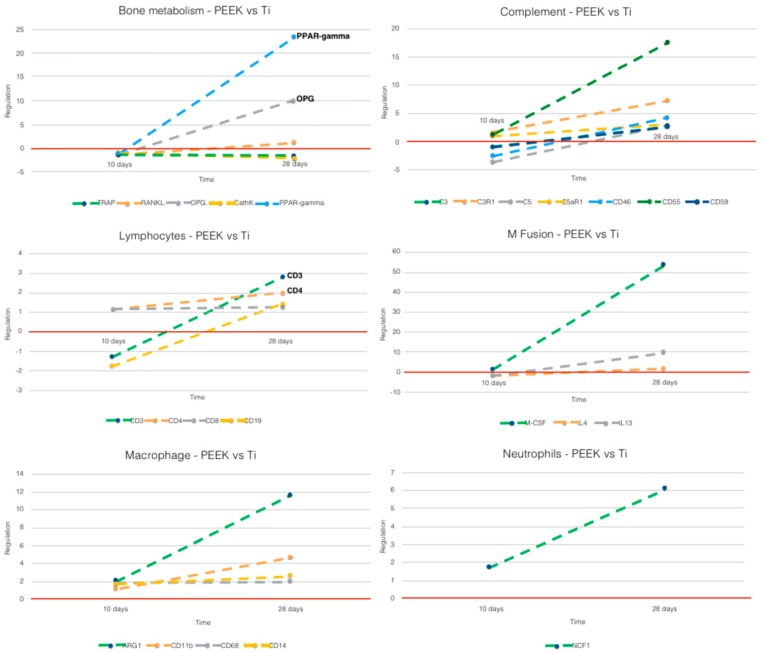
Comparative analysis of gene expression between 10 and 28 days for PEEK vs. Ti. Horizontal red line: zero regulation mark; x-axis: time; y-axis: gene marker regulation (10 or 28 days). Intermittent lines: do not represent actual results at time points other than 10 or 28 days, but only highlight trend from 10 to 28 days.

The comparison between Cu and PEEK when compared to Ti was divided by respective outcome (Figure 6 and Figure 7): macrophage, complement, neutrophils, lymphocytes, macrophage fusion and bone metabolism. Data from 10 days have been published in Part I [15] of this study, if with another control.

#### 3.2.1. Cu vs. Ti (Figure 6)

Macrophages: The immune activation was clearly higher around Cu than Ti at both time points. The M2-macrophage phenotype (*ARG1*) was reduced around Cu, but still upregulated when compared to Ti. The M1 markers (combination of *CD68*, *CD11b* and *CD14*) were upregulated slightly around Cu at both 10 and 28 days, i.e., Cu sustained a proinflammatory environment after the acute inflammatory and beginning of the bone remodeling period.

Complement: The results show that *C5* expression increased around Cu from 10 to 28 days, when compared to Ti (both *C5aR1* and *C5* suffered a sharp increase in regulation while the *CD59* (a *C5* inhibitor) was sharply downregulated over time). 

Neutrophils: There was a shift observed for *NCF1* from upregulation at 10 days to downregulation at 28 days. This likely indicates a change (reduction) in the presence of neutrophils around Cu.

Lymphocytes: The *CD4* reduction (from up- to downregulated) at 28 days around Cu may indicate a decrease in T_helper_/T_reg_ function, whereas effector T_cytotoxic_ (*CD8*) and B cells (*CD19*) remain slightly upregulated.

Macrophage fusion: More pronounced *IL4* downregulation and *IL13* upregulation from 10 to 28 days. 

Bone metabolism: The *RANKL/OPG* shunt reveals an obvious shift around Cu between 10 and 28 days. *RANKL* changes from upregulated to downregulated and the opposite for *OPG*, which becomes upregulated at 28 days, meaning a suppression of osteoclastogenesis from 10 to 28 days.

#### 3.2.2. PEEK vs. Ti (Figure 7)

It should be noted at 28 days that the results for PEEK should be read with caution since it was only possibility to retrieve mRNA from two of the five samples at 28 days. The possible reasons for this will be discussed below. Data from 10 days have been published in Part I [15] of this study, if with another control.

Macrophages: The macrophage activation around PEEK observed at 10 days and 28 days showed increase in both M1 and M2 markers. This confirms that there was an elevated M1 activation at 28 days, as well as a strong increase in M2-phenotype.

Complement: The results indicate, after 28 days, a continued immune activation around PEEK, especially pronounced for *C3*-related markers (*C3*, *C3aR1*, *CD46*, and *CD55*), and with slight upregulation for *C5*-related markers.

Neutrophils: *NCF1*, the specific neutrophil marker, was at both time points upregulated for PEEK, but showed a sharp increase at 28 days.

Lymphocytes: All lymphocyte markers increased from 10 to 28 days around PEEK. This was especially evident for *CD4*+ T_h/reg_ and *CD19*+ B cells.

Macrophage fusion: The results indicate a sharp increase in macrophage fusion markers around PEEK, also with a possible contribution to a M2-macrophage phenotype (*IL13*- confirmed by the above mentioned *ARG1* upregulation at 28 days). *M-CSF* also contributes to bone/adipose tissue balance in the osseous tissue, an important finding for PEEK and osseointegration in general, as discussed below.

Bone metabolism: The results suggest formation of adipose-like tissue around PEEK, as expressed by the extreme upregulation of *PPAR-ga*mma and by the upregulation of *M-CSF*. Suppression of bone resorption was sustained over time—RANKL still shows at 28 days some upregulation, but was overtaken by a sharp upregulation of *OPG*.

### 3.3. Histological Analysis

**Figure 8:**
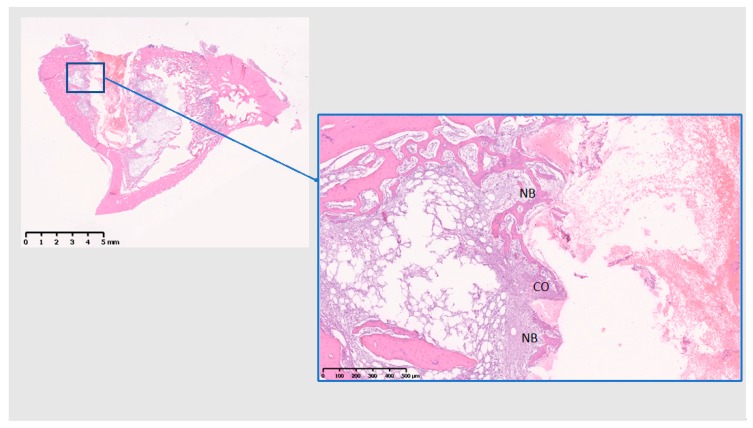
Histological analysis of Ti (10 days). NB: New bone; CO: Contact osteogenesis. Collagen proliferation and some initial calcification to form new bone in the threads. Scale bars: 5 mm and 500 µm.

**Figure 9:**
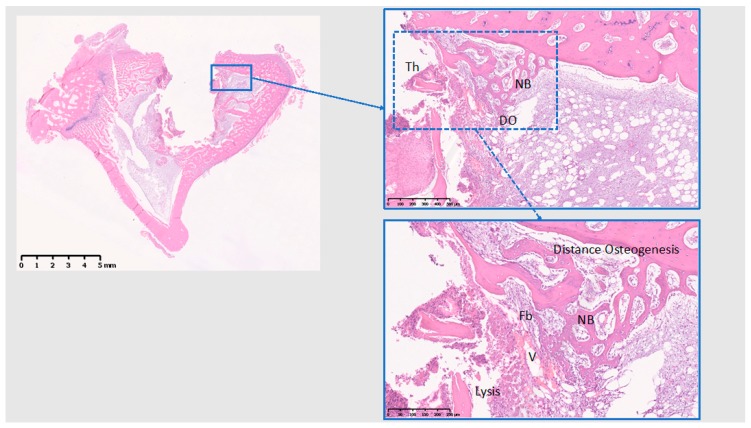
Histological analysis of Cu (10 days). Lytic area next to the implant; Fb: fibroproliferative; NB: New bone; DO: distance osteogenesis; Th: implant thread; V: blood vessel. New bone forming away from the implant surface. Scale bars: 5 mm, 500 µm, and 250 µm clockwise from left.

**Figure 10:**
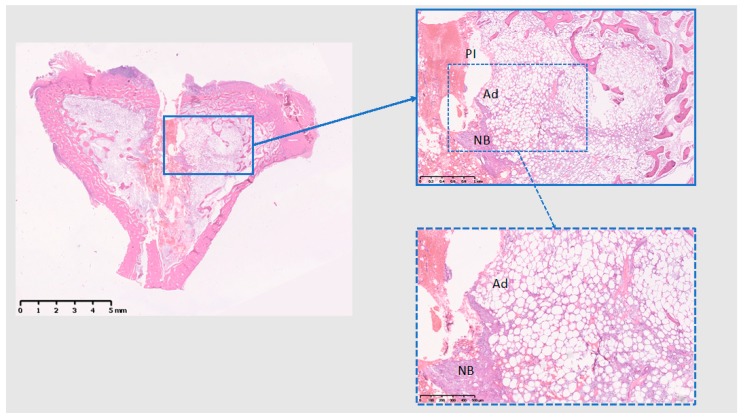
Histological analysis of PEEK (10 days). PI: PEEK implant; NB: New bone; Ad: Adipose tissue. Some collagen proliferation in one thread, adipose tissue also on the implant surface. Scale bars: 5 mm, 1 mm, and 500 µm.

The histological analysis was performed at tissue level. At 10 days (Figure 8, Figure 9 and Figure 10), Ti presents with initial bone formation within the threads, represented by unorganized collagen proliferation, whereas Cu presents mostly a lytic and cell infiltrate area on the implant surface, followed by a fibrous layer and finally a new bone formation, away from the surface. At 10 days, PEEK demonstrated very little initial bone tissue formation in some threads, but mostly adipose tissue surrounding the implant. Data from 10 days have been published in Part I [15] of this study, if with another control.

**Figure 11:**
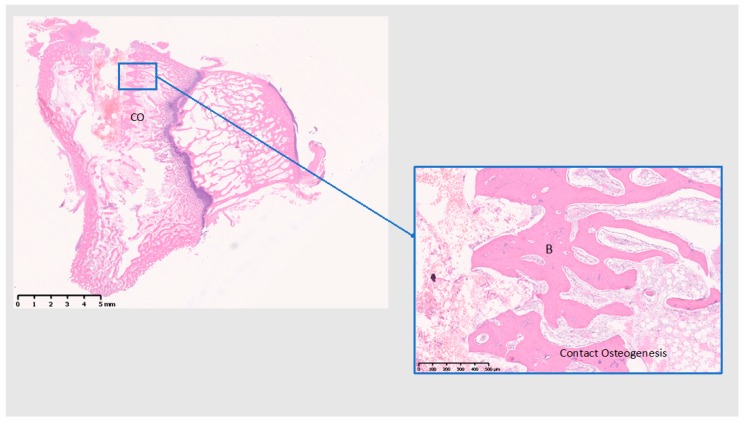
Histological analysis of Ti 28 (days). CO: contact osteogenesis. B: Bone. Formation of mature bone within the implant threads. Scale bars: 5 mm and 500 µm.

**Figure 12:**
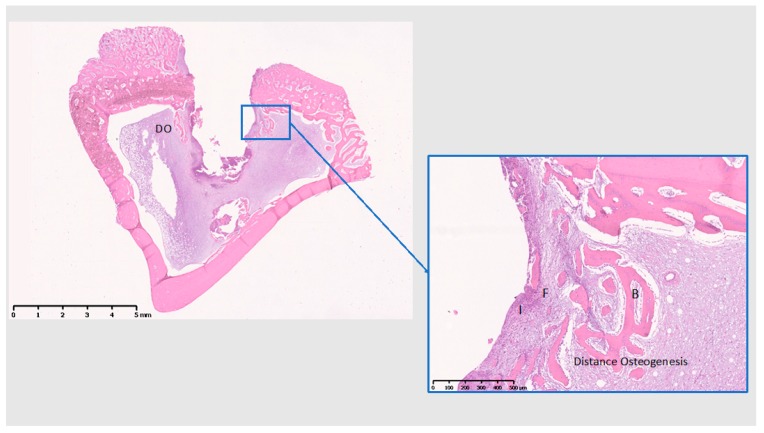
Histological analysis of Cu (28 days). DO: Distance osteogenesis; I: Inflammatory infiltrate; F: fibrous tissue; B: Bone. Formation of bone away from the implant surface, with a reduction of the infiltrate on the implant surface compared to 10 days. Scale bars: 5 mm and 500 µm.

**Figure 13:**
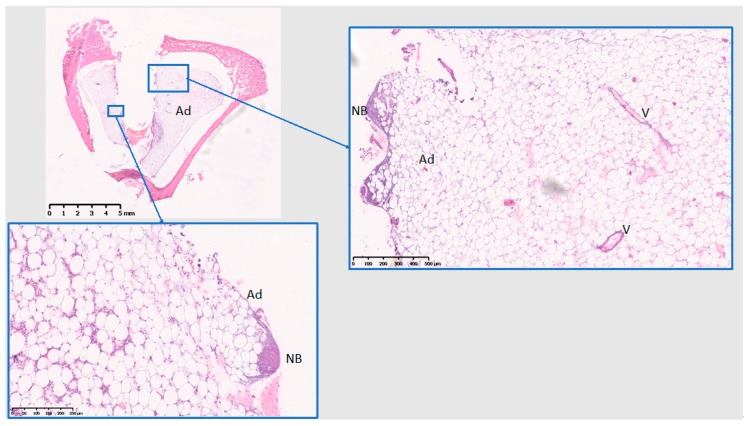
Histological analysis of PEEK (28 days). Ad: Adipose tissue; V: blood vessels; NB: New bone. Mostly adipose tissue proliferation and the bone tissue in the threads has not matured, nor calcified substantially. Scale bars: 5 mm, 500 µm, and 250 µm.

At 28 days (Figure 11, Figure 12 and Figure 13), Ti shows the bone within the threads maturing, while Cu demonstrates a reduction of the cell infiltrate, but still a bone formation away from the implant surface, whereas PEEK presents with mostly adipose tissue around the implant and the little bone tissue formed has not matured (unlike Ti) and shows little calcification.

## 4. Discussion

The osseointegration of materials for biomedical purposes has led to significant advances in patient treatment. Oral implants have become common in clinical practice, and by large base their success on osseointegration of materials such as titanium. Previous studies from the present authors have demonstrated the activation of the immune system around a material placed in bone [12,15], and it was hypothesized that the immune system has a regulatory function on the achievement of osseointegration [11]. In the present experimental study, the bone immune reaction around the materials polyetheretherketone (PEEK) and copper (Cu) was compared to titanium (Ti) as a control, at 10 and 28 days of implantation in rabbit tibia. The current study design aimed at comparing the immune modulation of two materials with poor osseointegration (Cu and PEEK) against a material that osseointegrates (Ti). The comparison between 10 and 28 days is important to understand the evolution of the reaction between the inflammatory period (10 days) and the postinflammatory period (28 days) of healing. Data from 10 days have been published in Part I [15] of this study, but with a Sham (no biomaterial) site as a control.

At 10 days, both PEEK and Cu showed upregulation of markers indicating a higher and different macrophage activity than was found around Ti (confirming the previous study [15]), namely predominantly an M2-phenotype, but also an elevated M1-phenotype. This was more pronounced around Cu than PEEK. At day 10, PEEK did not differ much from Ti, if with higher activation of the immune system (neutrophils and macrophages). This was however observed for Cu, with a higher overall immune activation. Both PEEK and Cu displayed some inhibition of bone resorption when compared to Ti. It is worth noting that PEEK, commonly referred to as a bioinert material [22,23], shows a higher immune activation than Ti at 10 days. 

After 28 days of implantation the scenario changes for both PEEK and Cu. Cu shows, as expected, a higher upregulation of the immune markers when compared to Ti, in all its innate components (complement, neutrophils, and macrophages of both M1- and M2-phenotypes). However, the macrophage fusion markers *IL-4* and *IL-13* expressions provide some contradictory indications since *IL-13* was upregulated and *IL-4* downregulated. This could be hypothesized as a stage for initial fusion into foreign body giant cells (FBGCs), but needs confirmation through further studies. Such macrophage fusion is not likely to be guided towards osteoclastogenesis, since bone resorption markers were widely downregulated, hence the macrophage behavior was probably directed towards the formation of FBGCs. However, *IL-13*, also known to induce the M2-phenotype [24] and combined with *ARG1* upregulation, confirms, at 28 days, the elevated M2 phenotype activity around Cu and PEEK compared to Ti, meaning a more pronounced host reparative effort for both materials, even if proinflammatory markers are simultaneously upregulated. The downregulation of bone resorption markers highlights the probable effort around Cu at 28 days, to build bone tissue around the implant for a bony delimitation that, as the histology shows, clearly develops away from the surface of the Cu implant. 

PEEK, on the other hand, seems to suffer a vast transformation at 28 days, into a high immune activation in the bone environment surrounding the implant, or rather fails to reduce that immune activation when compared to Ti. Reasons for the high immune upregulation around PEEK at 28 days are not well understood, although the current study results may offer an explanation regarding the bone/adipose tissue balance, as developed below. As mentioned in the results section, the 28 days results for PEEK should be read with caution, since only two subjects out of the five used for gene expression analysis actually enabled collection of enough mRNA to perform the PCR analysis. The difficulty to extract sufficient mRNA from the tissues surrounding PEEK implants was probably due to a low bone tissue formation adjacent to PEEK implants. Furthermore, the reasons behind the classical claim of a supposed bioinertness of PEEK is either that only in vitro studies of it have been presented or that in vivo studies have failed to analyze the immunological response; in contrast, the present results indicate immune activation around PEEK that may persist over extended periods of time. 

Regarding the comparison between the two time points of 10 and 28 days, for Cu, the *CD4* expression shifting over time from up- to downregulation, and the maintained upregulation of *CD8* and *CD19* at 28 days, demonstrates a shift in T_helper_/T_reg_ function whereas effector T_cytotoxic_ and B cells remain slightly upregulated over time. B cells, not only osteoblasts, are known to produce *OPG* in humans [25], which correlates with the increased gene expression of OPG at 28 days and adds another regulatory mechanism of the immune system on bone effector cells, and consequently on the ultimate anabolic/catabolic balance outcome of bone metabolism around implanted materials. It is important to mention that this B cell mechanism is known to be regulated by T cells, and the production of *OPG* by B/plasma cells can reach 64% of total *OPG* in some mammals [26], thus the present results highlight the immune regulation of bone metabolism around implanted materials. 

The notion that Cu starts to enter the remodeling phase and bone production at 28 days, even if at a distance as seen from the histological analysis, is further supported by the results for the above mentioned bone metabolism, with a sharp shift in *RANKL* (upregulated at 10 days and downregulated at 28 days) and in *OPG* (displaying the exact opposite trend) since *RANKL* induces osteoclastogenesis and *OPG* is the decoy molecule that stops this process, the results indicate a shift to a bone reparative environment around Cu at 28 days (through inhibition of the bone resorption inducive mechanisms). 

As for the results of the two time point comparisons between PEEK and Ti, the M1-macrophage activation at 28 days may impair bone formation at the PEEK implant surface, with a preferred fatty tissue deposition during repair, as indicated by the upregulation of *PPAR-gamma*, which is produced by differentiated macrophages [27] and in turn triggers the differentiation of adipocytes [28] at 28 days. The upregulation of complement around PEEK, the sharp increase in *NCF1* and the increase in regulation from 10 to 28 days around PEEK for Th/reg and B cells demonstrates that over time a higher immune activity is maintained around PEEK than Ti. This goes beyond the inflammatory period and is most likely proinflammatory. 

The upregulation at 28 days of macrophage fusion markers around PEEK indicates also other possible interpretations, such as the M2-phenotype connection of *IL-13* and the fact that *M-CSF*, besides its role in macrophage fusion into either osteoclasts or FBGCs, is intimately related to adipose tissue hyperplasia and growth (through proliferation) [29]. In the present study, the preferential adipose tissue growth observed on PEEK surface is supported at 28 days by the concomitant sharp upregulation of *PPAR-gamma* and *M-CSF*, and downregulation of *TRAP* and *Cathepsin-K* (bone resorption effectors), clearly indicating a sharp imbalance towards adipose tissue formation instead of bone formation around PEEK. It is important to note that in our previous study where Ti was compared to a Sham site at 28 days, no significant differences regarding *PPAR-gamma* or *M-CSF* were observed between the test and control [12], reinforcing the difference observed between PEEK and Ti at 28 days. Fat cell degeneration has previously been described in bone tissue after trauma upon overheating [30]. Such bone/adipose tissue imbalance, tilting towards more adipose tissue formation, has also been demonstrated in osteoporosis studies [31]. The present results after 28 days around PEEK support the description of this new-found mechanism for bone biomaterials. The orchestration of this process by the immune system has also been shown in literature [24], indicating a M1-macrophage chronic inflammation presence in proliferating adipose tissue [32], as well as *CD4*+ T_helper_/_reg_ and *CD19* B cells, as demonstrated in our results with a shift from downregulation at 10 days to upregulation at 28 days. The *PPAR-gamma* and *M-CSF* upregulation reaction likely overrules the OPG upregulation that would suppress bone resorption and increase osteoblast differentiation around PEEK; it is known that bone marrow mesenchymal stem cells (BMMSC) may either differentiate into osteoblasts or adipocytes [33], and PEEK, as demonstrated by the current results, seems to induce immune regulated adipocyte formation and proliferation in its vicinity. 

## 5. Conclusions

Overall, at 10 and 28 days after implantation in rabbit tibia, both Cu and PEEK show a higher immune activation than Ti. This more pronounced and extended immune reaction translates into a prolonged inflammatory phase of the healing period, and may be the cause for the bone tissue failing to form a layer in direct contact with these materials, as shown in the histological sections.

The current results demonstrate that, over time, different materials elicit a different immune regulation of bone metabolism around implanted materials. 

From a clinical orofacial perspective, it is fair to state that a fibrous tissue encapsulation or adipose instead of bone tissue formation could also occur around clinically placed titanium implants, should less ideal host conditions be present.

The results from the current study suggest that osseointegration may fail by at least two immunologically regulated mechanisms: (1) soft tissue encapsulation or (2) an imbalance in bone/adipose tissue formation around the implanted material.

## Figures and Tables

**Table 1:** Gene sequences.

Primer	Forward Sequence	Reverse Sequence	Accession No./Transcript ID
*NCF-1*	TTCATCCGCCACATTGCCC	GTCCTGCCACTTCACCAAGA	NM_001082102.1
*CD68*	TTTCCCCAGCTCTCCACCTC	CGATGATGAGGGGCACCAAG	ENSOCUT00000010382
*CD11b*	TTCAACCTGGAGACTGAGAACAC	TCAAACTGGACCACGCTCTG	ENSOCUT00000001589
*CD14*	TCTGAAAATCCTGGGCTGGG	TTCATTCCCGCGTTCCGTAG	ENSOCUT00000004218
*ARG1*	GGATCATTGGAGCCCCTTTCTC	TCAAGCAGACCAGCCTTTCTC	NM_001082108.1
*IL-4*	CTACCTCCACCACAAGGTGTC	CCAGTGTAGTCTGTCTGGCTT	ENSOCUT00000024099
*IL-13*	GCAGCCTCGTATCCCCAG	GGTTGACGCTCCACACCA	ENSOCUT00000000154
*M-CSF*	GGAACTCTCGCTCAGGCTC	ACATTCTTGATCTTCTCCAGCAAC	ENSOCUT00000030714
*OPG*	TGTGTGAATGCGAGGAAGGG	AACTGTATTCCGCTCTGGGG	ENSOCUT00000011149
*RANKL*	GAAGGTTCATGGTTCGATCTGG	CCAAGAGGACAGGCTCACTTT	ENSOCUT00000024354
*TRAP*	TTACTTCAGTGGCGTGCAGA	CGATCTGGGCTGAGACGTTG	NM_001081988.1
*CathK*	GGAACCGGGGCATTGACTCT	TGTACCCTCTGCATTTGGCTG	NM_001082641.1
*PPAR-* *γ*	CAAGGCGAGGGCGATCTT	ATGCGGATGGCGACTTCTTT	NM_001082148.1
*C3*	ACTCTGTCGAGAAGGAACGGG	CCTTGATTTGTTGATGCTGGCTG	NM_001082286.1
*C3aR1*	CATGTCAGTCAACCCCTGCT	GCGAATGGTTTTGCTCCCTG	ENSOCUT00000007435
*CD46*	TCCTGCTGTTCACTTTCTCGG	CATGTTCCCATCCTTGTTTACACTT	ENSOCUT00000033915
*CD55*	TGGTGTTGGGTGGAGTGACC	AGAGTGAAGCCTCTGTTGCATT	ENSOCUT00000031985
*CD59*	ACCACTGTCTCCTCCCAAGT	GCAATCTTCATACCGCCAACA	NM_001082712.1
*C5*	TCCAAAACTCTGCAACCTTAACA	AAATGCTTTGACACAACTTCCA	ENSOCUT00000005683
*C5aR1*	ACGTCAACTGCTGCATCAACC	AGGCTGGGGAGAGACTTGC	ENSOCUT00000029180
*CD3*	CCTGGGGACAGGAAGATGATGAC	CAGCACCACACGGGTTCCA	NM_001082001.1
*CD4*	CAACTGGAAACATGCGAACCA	TTGATGACCAGGGGGAAAGA	NM_001082313.2
*CD8*	GGCGTCTACTTCTGCATGACC	GAACCGGCACACTCTCTTCT	ENSOCUT00000009383
*CD19*	GGATGTATGTCTGTCGCCGT	AAGCAAAGCCACAACTGGAA	ENSOCUT00000028895
*GAPDH*	GGTGAAGGTCGGAGTGAACGG	CATGTAGACCATGTAGTGGAGGTCA	NM_001082253.1
*ACT-* *β*	TCATTCCAAATATCGTGAGATGCC	TACACAAATGCGATGCTGCC	NM_001101683.1
*LDHA*	TGCAGACAAGGAACAGTGGA	CCCAGGTAGTGTAGCCCTT	NM_001082277.1

*NCF-1* (neutrophil cytosolic factor 1); *CD68* (macrosialin); *CD11b* (*MAC-1*, macrophage marker); *CD14* (monocyte differentiation antigen *CD14*); *ARG1* (Arginase 1); *IL-4* (Interleukin 4); *IL-13* (Interleukin 13); *M-CSF* (colony stimulating factor-macrophage); *OPG* (osteoprotegerin); *RANKL* (Receptor activator of nuclear factor kappa-B ligand); *TRAP* (tartrate resistant acid phosphatase); *CathK* (cathepsin K); *PPAR-γ* (peroxisome proliferator activated receptor gamma); *C3* (complement component 3); *C3aR1* (complement component 3a receptor 1); *CD46* (complement regulatory protein); *CD55* (decay accelerating factor for complement); *CD59* (complement regulatory protein); *C5* (complement component 5); *C5aR1* (complement component 5a receptor 1); *CD3* (T cell surface glycoprotein CD3); *CD4* (T cell surface glycoprotein CD4); *CD8* (T cell transmembrane glycoprotein CD8); *CD19* (B-lymphocyte surface protein CD19); *GAPDH* (glyceraldehyde-3-phosphate dehydrogenase); *ACT-β* (actin beta); *LDHA* (lactate dehydrogenase A).

**Table 2:** Correspondence between studied gene expression and biological entities.

Biological Entity	Gene
Neutrophil	*NCF-1*
Macrophage	*CD68*, *CD11b*, *CD14*, *ARG1*
Macrophage fusion	*IL-4*, *IL-13*, *M-CSF*
Bone resorption	*OPG*, *RANKL*, *TRAP*, *CathK*, *PPAR-γ*
Complement	Activation: *C3*, *C3aR1*, *C5*, *C5aR1*; Inhibition: *CD46*, *CD55*, *CD59*
T lymphocytes	*CD3*, *CD4*, *CD8*
B-lymphocytes	*CD19*
Reference genes	*GAPDH*, *ACT-β*, *LDHA*

*NCF-1* (neutrophil cytosolic factor 1); *CD68* (macrosialin); *CD11b* (*MAC-1*, macrophage marker); *CD14* (monocyte differentiation antigen *CD14*); *ARG1* (Arginase 1); *IL-4* (Interleukin 4); *IL-13* (Interleukin 13); *M-CSF* (colony stimulating factor-macrophage); *OPG* (osteoprotegerin); *RANKL* (Receptor activator of nuclear factor kappa-B ligand); *TRAP* (tartrate resistant acid phosphatase); *CathK* (cathepsin K); *PPAR-γ* (peroxisome proliferator activated receptor gamma); *C3* (complement component 3); *C3aR1* (complement component 3a receptor 1); *CD46* (complement regulatory protein); *CD55* (decay accelerating factor for complement); *CD59* (complement regulatory protein); *C5* (complement component 5); *C5aR1* (complement component 5a receptor 1); *CD3* (T cell surface glycoprotein CD3); *CD4* (T cell surface glycoprotein CD4); *CD8* (T cell transmembrane glycoprotein CD8); *CD19* (B-lymphocyte surface protein CD19); *GAPDH* (glyceraldehyde-3-phosphate dehydrogenase); *ACT-β* (actin beta); *LDHA* (lactate dehydrogenase A).

**Table 3:** Gene expression analysis of Cu compared to Ti (10 days).

Marker	Regulation	*p* Value
PPAR-G	–1.72	0.087477
TRAP	–1.98	0.137344
OPG	–2.03	0.539750
CD3	–1.52	0.411951
IL-4	–1.71	0.185082
ARG1	14.17	0.006031
NCF1	1.96	0.125414
C3aR1	1.66	0.154634
CD14	1.50	0.414019
CD4	1.53	0.431624
CD19	1.64	0.279592

Minus values: downregulation; plus values: upregulation.

**Table 4:** Gene expression analysis of PEEK compared to Ti (10 days).

Marker	Regulation	*p* Value
PPAR-G	–1.57	0.550176
TRAP	–1.68	0.008821
OPG	–1.70	0.089695
CD19	–2.14	0.111496
IL-4	–2.14	0.251881
ARG1	1.71	0.361937
NCF1	1.50	0.333874
CD68	1.62	0.556273

Minus values: downregulation; plus values: upregulation.

**Table 5:** Gene expression analysis of Cu compared to Ti (28 days).

Marker	Regulation	*p*-Value
*C3*	–4.64	0.016332
*CD59*	–4.93	0.073238
*RANKL*	–2.96	0.049318
*PPAR-G*	–2.29	0.115578
*TRAP*	–1.49	0.036164
*CATH-K*	–1.70	0.039611
*CD3*	–2.22	0.073334
*CD4*	–1.69	0.132057
*IL-4*	–3.43	0.379695
*ARG1*	7.69	0.007955
*CD14*	2.09	0.260868
*CD68*	2.35	0.473322
*C5aR1*	3.03	0.080240
*C3aR1*	2.25	0.084210
*IL-13*	3.84	0.051296

Minus values: downregulation; plus values: upregulation.

**Table 6:** Gene expression analysis of PEEK compared to Ti (28 days).

Marker	Regulation	*p*-Value
*TRAP*	–2.09	0.112708
*CATHK*	–2.31	0.111423
*CD55*	18.29	0.000000
*C3aR1*	8.31	0.000818
*C5aR1*	3.46	0.002609
*CD46*	4.68	0.035842
*CD59*	3.03	0.052696
*ARG1*	18.72	0.000011
*CD11b*	3.95	0.006903
*CD14*	2.14	0.020201
*NCF-1*	7.04	0.001291
*CD3*	2.60	0.014589
*CD4*	3.28	0.006753
*CD8b*	1.53	0.393394
*CD19*	1.30	0.182925
*MCSF*	57.55	0.000000
*IL-13*	8.11	0.024702
*PPAR-G*	25.54	0.000000
*OPG*	13.77	0.000687

Only 2 subjects to be interpreted with caution. Minus values: downregulation; plus values: upregulation.
